# The Effect of Ozone Therapy on Psoriasis

**DOI:** 10.1002/ccr3.72021

**Published:** 2026-05-23

**Authors:** Maryam Aghaei, Shahrzad Aghaei, Zabihollah Shahmoradi, Mohammad‐Ali Nilforoushzadeh, Seyed Hossein Hejazi

**Affiliations:** ^1^ Skin Diseases and Leishmaniasis Research Center Isfahan University of Medical Sciences Isfahan Iran; ^2^ Department of Molecular Medicine School of Advanced Technologies, Shahrekord University of Medical Sciences Shahrekord Iran; ^3^ Skin Diseases and Leishmaniasis Research Center, Department of Dermatology Isfahan University of Medical Sciences Isfahan Iran; ^4^ Skin and Stem Cell Research Center Tehran University of Medical Sciences Tehran Iran; ^5^ Skin Diseases and Leishmaniasis Research Center, Department of Parasitology and Mycology School of Medicine, Isfahan University of Medical Sciences Isfahan Iran

**Keywords:** ozonate olive oil, ozone sauna, ozone therapy, psoriasis

## Abstract

Psoriasis is an immune‐mediated skin disease in which genetic and environmental factors have a significant role. Olive oil with antioxidant properties is an effective adjunct treatment for skin diseases. Furthermore, ozone by introducing O_2_ into the bloodstream controls the response of cell‐mediated immunity and leads to improve disease. So, the aim of this study was to evaluate the efficacy of ozone therapy in the treatment of Psoriasis. Topical therapy of lesions of a man suffering from psoriasis was carried out using ozonate olive oil two times a day and ozone sauna once a week during a month. Patient showed considerable improvement after about twenty days. As the itching and silvery‐white scaling decreased, the lesions began to resolve after three weeks. Furthermore, no recurrence was noted after 3 months of follow‐up. It is suggested that ozone therapy, in appropriate formulations and in controlled cases, has a role in the treatment of patients with psoriasis by inhibiting the inflammatory pathways and prompting regenerative characteristics, without significant adverse effects and the necessity for systemic pharmacological agents. So, more studies on a greater population are needed to confirm this finding.

## Key Clinical Message

Topical application of ozonated olive oil combined with ozone sauna therapy may serve as an effective adjunct treatment for psoriasis, reducing itching and scaling while minimizing the need for systemic drugs. Larger clinical studies are needed to confirm these findings.

## 1 Introduction

Psoriasis is an autoimmune inflammatory skin disease. This disease is chronic with a tendency to exacerbations and remissions, and is not contagious. The disease affects 2%–3% of the population with a higher incidence in females than males. Although it affects children and older people, the onset of the disease appears commonly at about twenty years old. It is characterized by discrete patches surmounted by characteristic silvery scaling [1]. These skin lesions are typically pink, itchy, and scaly that affect sites such as the scalp, tips of fingers and toes, palms, soles, umbilicus, gluteus, under the breasts and genitals, elbows, knees, shins, and sacrum [2]. There are various types of psoriasis, including psoriatic arthritis, scalp psoriasis, flexural psoriasis, guttate psoriasis, pustular psoriasis, nail psoriasis, erythrodermic psoriasis, plaque psoriasis [1]. Plaque psoriasis is the most common form and affects 80% to 90% of people with psoriasis. These plaques are circular‐to‐oval red with silvery white scales on top and frequently affect the skin of the elbows and knees [3].

The cause of psoriasis is unknown, but it is believed that psoriasis is an autoimmune disease in which genetic and environmental factors have a significant role. Normally, the skin cells mature and are shed from the skin's surface every 28 to 30 days [4]. But when psoriasis develops, T cells become activated and migrate to the dermis, triggering the release of cytokines like tumor necrosis factor‐alpha, which leads to the inflammation and accelerates the growth of skin cells. As skin cells mature in 3 to 6 days, they move to the skin surface and instead of being shed, the skin cells pile up, leading to the characteristic clinical features of scaling and redness [5]. Several factors are also thought to aggravate psoriasis, such as stress, infections, certain medications, changes in climate, alcohol consumption, smoking, obesity, and family history [6].

The diagnosis of psoriasis is clinical, and no special blood tests or diagnostic procedures are required to diagnose psoriasis. However, a skin biopsy may be performed to confirm the diagnosis [7].

Therapeutic agents that either modulate the immune system or normalize the differentiation process of psoriatic keratinocytes are suggested for treating psoriasis. There are topical agents (emollients, dithranol, tar, deltanoids, corticoids, tacrolimus) for mild form of disease, phototherapy for form of moderate disease, systemic agents (methotrexate, cyclosporin, acitrecin, hydroxyurea, fumarates) for severe form of disease, and homeopathic approach which can help to control the symptoms [1]. Moreover, herbal remedies (as cream or extract or oil) for psoriasis are increasingly popular. The herbs include *Aloe vera* , Chamomile, Lavender, Licorice root, Yarrow, Oatmeal, Almond have anti‐inflammation, itching and pain properties [8]. Although, orthodox medicine has a variety of anti‐infective agents, unfortunately some of them are expensive and not so useful because of drug‐resistant pathogens and side effects. Recently, ozone therapy has been recognized as useful treatment. Ozone therapy for psoriasis exerts both analgesic and anti‐inflammatory effects. As, ozone oxidative neutralizes chemical inflammation mediators such as histamine, cytokines and phospholipase A2, and blocks the nociceptors responsible for pain perception. Ozone therapy regulates cellular immunity, modulates epidermal cell growth, and inhibits infection in skin lesions. Furthermore, it increases tissue oxygenation via improved microcirculation, induces cellular metabolism including ATP production, and stimulates growth factors [9]. With regard to therapeutic effect of ozone therapy, we studied one man patient for searching effect of ozonate olive oil and ozone Suna on psoriasis.

## 2 Case History/Examination

The case was a 43‐year‐old male from Iran with manifestations of multiple itchy, white‐silvery, scaly patches measuring more than 4–6 cm in diameter, located on the shoulders, middle, and extremities. The lesions had been present for ten years and had gradually increased in size and number. The palms, soles, and nails were uninvolved. There was a history of itching or sudden exacerbation with stress, medication, or toast meat. He had a medical history of skin diseases such as psoriasis, rosacea, and vitiligo among his second‐degree relatives. The case had been treated with emollients and topical steroid by a few dermatologists, but the lesions did not resolve. In the present study, written informed consent to participate and publish all clinical and imaging data was obtained from the participant.

## 3 Differential Diagnosis

The diagnosis of psoriasis was made based on the characteristic clinical features, including well‐demarcated erythematous plaques with white‐silvery scales, a chronic relapsing course, and positive family history. Other possible conditions such as eczema and lichen planus were clinically excluded by a board‐certified dermatologist. No laboratory or immunological tests were performed due to the classic appearance of the psoriasis lesions.

## 4 Topical Ozonated Olive Oil and Ozone Sauna Application

The ozone sauna system (Gardina Company, Tehran, Iran) consisted of a cabin made of plastic laminate material, with an internal volume of approximately 440 L. Ozone‐enriched oxygen (O_2_–O_3_ mixture) was generated by an ozone device equipped with a photometric O_3_ concentration controller and was continuously circulated through the cabin at a rate of 1 L/min. The ozone concentration was progressively increased from 15 to 25 μg/mL throughout the treatment sessions. Steam was produced by a thermostatically controlled heating element set at 90°C, maintaining the cabin temperature between 46°C and 50°C throughout the session. Each treatment session lasted approximately 20 min. At the end of each session, the gas flow was stopped and the residual gas was quickly evacuated. Subsequently, the participant was instructed to take a shower immediately. Ozone sauna therapy was performed once per week. Moreover, the participant was treated with topical ozonated oil [10], twice a day. Both treatments were administered concurrently over the same one‐month period.

## 5 Conclusion and Results

**FIGURE 1:**
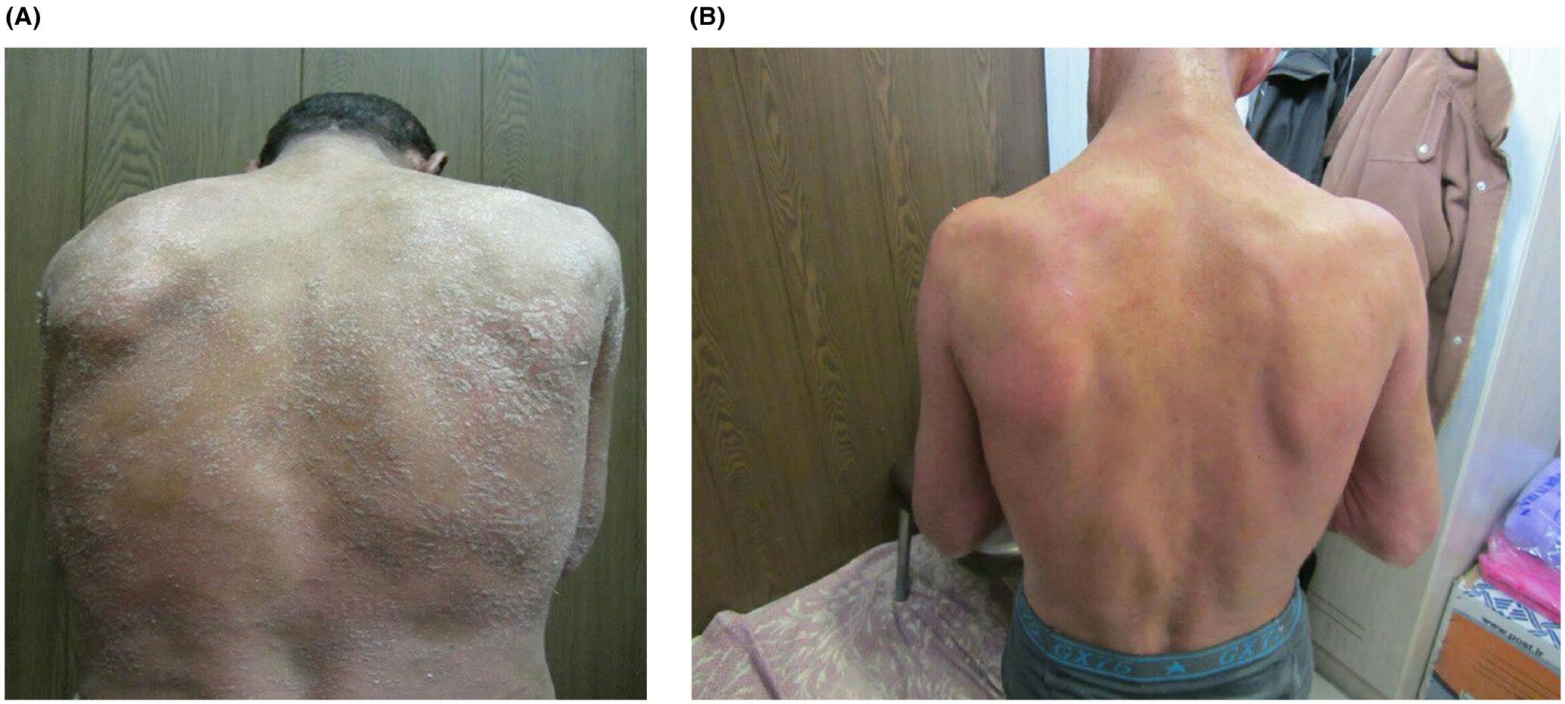
Improvement of psoriasis lesions in a patient. Before (A) and after (B) treatment with ozonated olive oil and ozone sauna.

To decrease itchy and white‐silvery scaling were considered as positive effects of method. Noticeable improvement was observed after three weeks of treatment, with a significant reduction of erythema, scaling, and itching. After one month, most lesions had resolved (Figure 1), and there were no recurrences or new lesions during the three‐month follow‐up period.

## 6 Discussion

In this study for the first time, the effect of ozonated olive oil and ozone sauna on psoriasis was surveyed and results showed distinct improvement of the disease without significant adverse effects.

Psoriasis as a chronic inflammatory skin disease does not have effective treatment options, but ozonated oils have been suggested as an alternative to treat it. These oils have an active molecule in their chemical structure that has been widely used to treat more than 50 diseases, including skin diseases and hypersensitivity [11]. For the treatment of psoriasis, olive oil has shown equivalent and, in some parameters, superior efficacy to topical corticosteroid preparations. This efficacy can be explained by the ability of olive oil to induce keratinocyte differentiation through enhanced transcription of the KRT10 gene, mediated by tumor protein Tp63, leading to clinical symptoms improvement of psoriasis [12]. Furthermore, ozone therapy exerts anti‐inflammatory and antioxidant effects through decreasing pro‐inflammatory cytokines and enhancing antioxidant enzyme activity. In addition, ozone modulates the immune response by downregulating T lymphocytes activation and promoting tissue oxygenation through improved microcirculation [9].

The clinical improvement observed in this patient may be attributed to several biological and antimicrobial properties of olive oil and ozone [13]. These combined effects may contribute to the reduction in erythema, scaling, and itching observed after treatment with ozonated olive oil and ozone Suna in the present case, after three weeks. As after one month, most lesions had resolved, and no recurrence was seen after three months of follow‐up.

In agreement with our results, Al‐Waili et al. [14] investigated topical application of natural honey, beeswax and olive oil mixture for psoriasis in the clinical trial. Results showed that honey mixture appears useful in the management of psoriasis vulgaris compared to standard medicine (betamethasone, clobetasol). In a similar study, Zeng et al. [15] showed that ozone therapy reduces the local inflammatory response mediated by NF‐κB and the activation of Th17 cells in the treatment of psoriasis. Recio et al. [16] also revealed the positive biological effects of ozone when it was administered rectally in psoriasis patients. As principal results were achieved in the female group, with 65% of recovery and signs such as pruritus and erythema disappeared. In addition, Abdulsalam et al. [17], showed ozonated olive oil is an effective mode of treatment in IMQ‐induced psoriasis when compared to olive oil alone in mice, through modulation of NF‐κB signaling pathways, leading to a reduction in inflammatory cytokines.

Furthermore, it is suggested that the Mediterranean diet, which includes the use of olive oil, has systemic effects on psoriasis through reducing cytokine levels and inflammation [18]. The use of supplements as an adjunctive treatment for psoriasis is also suitable. As the results of a pilot study by Acosta et al. [19] showed that a cosmetic and nutritional product based on olive phenols was able to reduce skin manifestations in patients with moderate psoriasis and did not cause side effects. In contrast, Donato‐Trancoso et al. [20] evaluated the effect mechanisms of olive oil on psoriasis in vivo. They found that dietary intake of olive oil aggravates the symptoms of psoriatic skin lesions in mice through the overexpression of Nrf2 and an imbalance in polyunsaturated fatty acids levels such as oleic and linoleic, suggesting that a diet rich in olive oil may have significant negative effects on psoriasis.

Collectively, the results of the aforementioned studies show that ozone therapy is effective in the treatment of psoriasis when used in appropriate formulations and under controlled conditions, making it a promising therapeutic alternative for psoriasis.

## Author Contributions

**Maryam Aghaei:** investigation, methodology, writing – original draft, writing – review and editing. **Shahrzad Aghaei:** writing – original draft. **Zabihollah Shahmoradi:** writing – review and editing. **Seyed Hossein Hejazi:** writing – review and editing. **Mohammad‐Ali Nilforoushzadeh:** writing – review and editing.

## Funding

The authors have nothing to report.

## Consent

The authors certify that they have obtained all appropriate patient consent forms. In the form, the patient has given his consent for his images and other clinical information to be reported in the journal. The patient understands that his name and initial will not be published, and due efforts will be made to conceal his identity, but anonymity cannot be guaranteed.

## Conflicts of Interest

The authors declare no conflicts of interest.

## Data Availability

The data set supporting the conclusions is available upon request to the corresponding author.
